# Sonogenetics is a non-invasive approach to activating neurons in *Caenorhabditis elegans*

**DOI:** 10.1038/ncomms9264

**Published:** 2015-09-15

**Authors:** Stuart Ibsen, Ada Tong, Carolyn Schutt, Sadik Esener, Sreekanth H. Chalasani

**Affiliations:** 1 Molecular Neurobiology Laboratory, The Salk Institute for Biological Studies, La Jolla, California 92037, USA; 2 Department of Bioengineering, Moores Cancer Center, University of California San Diego, La Jolla, California 92093, USA; 3 Department of Nanoengineering, Moores Cancer Center, University of California San Diego, La Jolla, California 92093, USA

## Abstract

A major challenge in neuroscience is to reliably activate individual neurons, particularly those in deeper brain regions. Current optogenetic approaches require invasive surgical procedures to deliver light of specific wavelengths to target cells to activate or silence them. Here, we demonstrate the use of low-pressure ultrasound as a non-invasive trigger to activate specific ultrasonically sensitized neurons in the nematode, *Caenorhabditis elegans*. We first show that wild-type animals are insensitive to low-pressure ultrasound and require gas-filled microbubbles to transduce the ultrasound wave. We find that neuron-specific misexpression of TRP-4, the pore-forming subunit of a mechanotransduction channel, sensitizes neurons to ultrasound stimulus, resulting in behavioural outputs. Furthermore, we use this approach to manipulate the function of sensory neurons and interneurons and identify a role for PVD sensory neurons in modifying locomotory behaviours. We suggest that this method can be broadly applied to manipulate cellular functions *in vivo*.

## Abstract (web summary)

Common optogenetic approaches require surgical procedures to deliver light of specific wavelengths to the target cells. Here the authors demonstrate the use of low-pressure ultrasound as a non-invasive trigger to activate specific neurons in *Caenorhabditis elegans* and find that the mechanotransduction channel TRP-4 sensitizes cells to the ultrasound stimulus.

## Graphical abstract

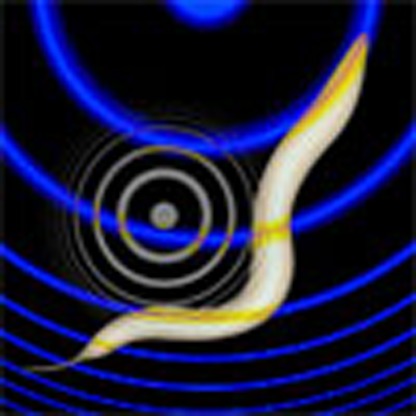

Understanding how neural circuits generate specific behaviours requires identifying the participating neurons and subsequently recording and *perturbing* their activity. The best-understood motor circuit, the crab stomatogastric ganglion, has benefited from electrophysiological access to well-defined cell types as well as an ability to manipulate their activity^1^. A number of approaches have been developed for manipulating neuronal activity using light (optogenetics) or small molecules^2^,^3^. While these methods have revealed insights into circuit computations in a variety of model systems, they suffer from one drawback: difficulty in delivering stimulus to target neurons located in deeper brain regions^4^,^5^. To address this issue, we have developed a new method that genetically sensitizes targeted neurons to low-pressure ultrasound, a stimulus that can be delivered to small regions of deep tissue throughout an animal, including its brain.

Ultrasound stimulation is non-invasive. This is particularly important for manipulating vertebrate neurons, as it eliminates the need for invasive surgery to insert fibre optics (required for some current optogenetic methods^6^). Furthermore, ultrasound is well-suited for stimulating neuron populations as it focuses easily through intact thin bone and deep tissue^7^ to volumes of a few cubic millimetres^8^,^9^. Previously, ultrasound had been used to directly stimulate clusters of neurons *in vitro* or within the brains of several model organisms^10^,^11^,^12^,^13^,^14^,^15^. Interestingly, activating neurons in these cases requires exposure to continuous or repeated pulses of ultrasound between 690 kHz and 3 MHz^16^. Ultrasound has also been shown to safely manipulate deep nerve structures in human hands to reduce chronic pain^17^. Despite these observations and the development of theoretical models^18^, the mechanisms by which ultrasound stimulates these neurons remain poorly understood. Moreover, an additional challenge lies in developing a method to target individual neurons to ultrasound stimulation, as the minimum focal zone of the ultrasound is larger than an individual cell.

To overcome these challenges we developed a new method to stimulate neurons that we call ‘sonogenetics’, using the nematode, *Caenorhabditis elegans. C. elegans* with its small nervous system consisting of just 302 neurons connected by identified synapses^19^ has well-characterized robust behaviours^20^ and reliable methods to monitor neural activity^21^. We identified a pore-forming subunit of a mechanotransduction channel, TRP-4, that is sensitive to low-pressure ultrasound. Further, we show that individual neurons misexpressing TRP-4 show changes in neural activity upon low-pressure ultrasound stimulation. Finally, we correlate these neural activity changes with specific behaviours at the level of whole animals.

## Results

### Imaging set-up delivers ultrasound waves to animals

**Figure 1:**
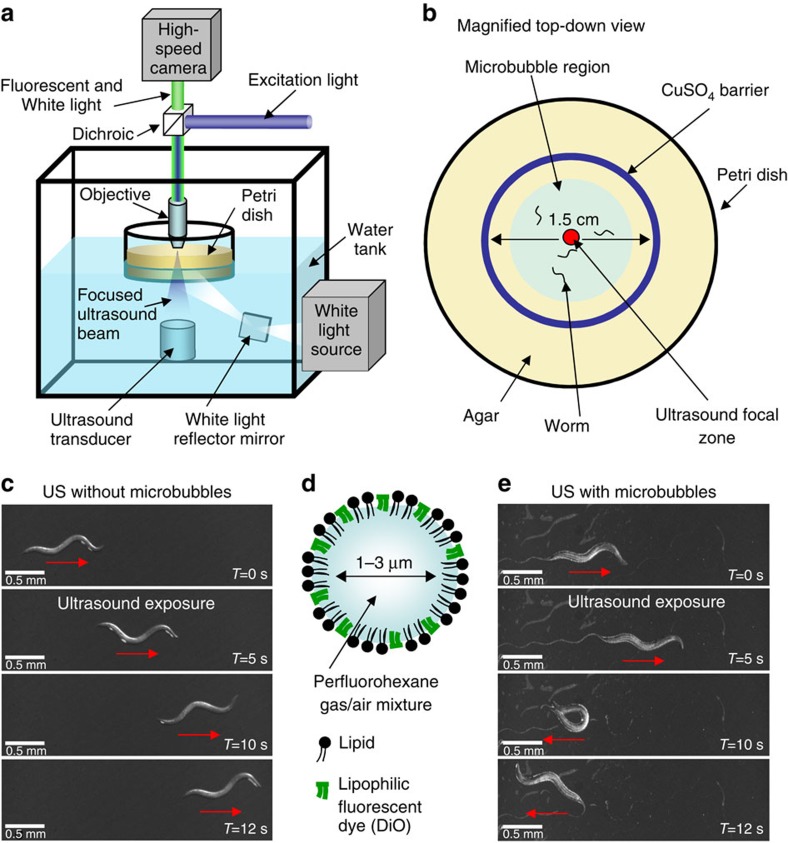
Amplifying ultrasound signals using microbubbles modifies animal behaviour. (**a**) Schematic of the computer-controlled imaging and ultrasound exposure system (frontal view) and (**b**) the agar plate with animals (top view) corralled into a small area by a copper barrier (1.5 cm in diameter). (**c**) Image sequence showing that animals do not respond to low-pressure ultrasound (US) alone. (**d**) Schematic of a stabilized microbubble. (**e**) Images showing that animals exhibit reversals and omega bends upon ultrasound (US) stimulus (single 10-ms pulse, 2.25 MHz with peak negative pressure of 0.9 MPa) in the presence of microbubbles.

To investigate the role of ultrasound in wild-type *C. elegans* neural activity and behaviour, we developed a set-up that aligns optical imaging with the ultrasound focal zone (Fig. 1a). Ultrasound with different peak negative pressures was generated from a transducer and focused onto an agar plate, where animals were corralled into a small area using a copper solution (Fig. 1b). The transducer focused the ultrasound wave to a 1-mm-diameter circular area at the agar surface (red circle in Fig. 1b). The entire set-up was placed in a large tank filled with water to facilitate uniform transduction of the ultrasound wave (Fig. 1a). Depending on solution or tissue gas concentrations, high ultrasound peak negative pressures (>2.5 MPa) can create inertial cavitation, with the resulting shockwaves compromising the integrity of cell membranes^22^,^23^. Consistently, we observed that animals exposed to multiple pulses of high ultrasound pressures were unable to maintain their normal body posture (Supplementary Fig. 1). Therefore, we chose to use low-pressure ultrasound, which does not cause these damaging effects, to stimulate animal behaviour.

We found that a single 10-ms-duration ultrasound pulse of 2.25 MHz and peak negative pressures below 0.9 MPa had no effect on animal behaviour (Fig. 1c). The mechanical disturbances^24^ of the fluid and tissue in the ultrasound focal zone take the form of compression and expansion deformations as well as bulk tissue distortions caused by acoustic radiation forces, but at low pressures they were not large enough to influence *C. elegans* locomotion. Previous studies have shown that ultrasound waves can cause temperature changes in the focal zone^25^. We first estimated the temperature increase as a result of ultrasound exposure. In a previous study, a continuous 1.1-MHz-ultrasound pulse with a peak negative pressure of 2.6 MPa increased the temperature of the surrounding media at the rate of 35 °C s^−1^ (ref. 25). Using these data, we estimated the temperature increase around the worms on the agar surface to be 0.04 °C for a single ultrasound pulse at 0.9 MPa. Moreover, we also directly measured the magnitude of temperature change on the agar surface using a miniature thermocouple (Supplementary Fig. 2) and found that an ultrasound peak negative pressure of 0.7 MPa caused a temperature increase of less than 0.1 °C (see Methods). This is a temperature stimulus that animals including *C. elegans* are unlikely to detect^26^,^27^. Together, these results show that *C. elegans* is unlikely to respond to the temperature and mechanical changes induced by the low-pressure ultrasound wave.

### Microbubbles amplify ultrasound mechanical deformations

**Figure 2:**
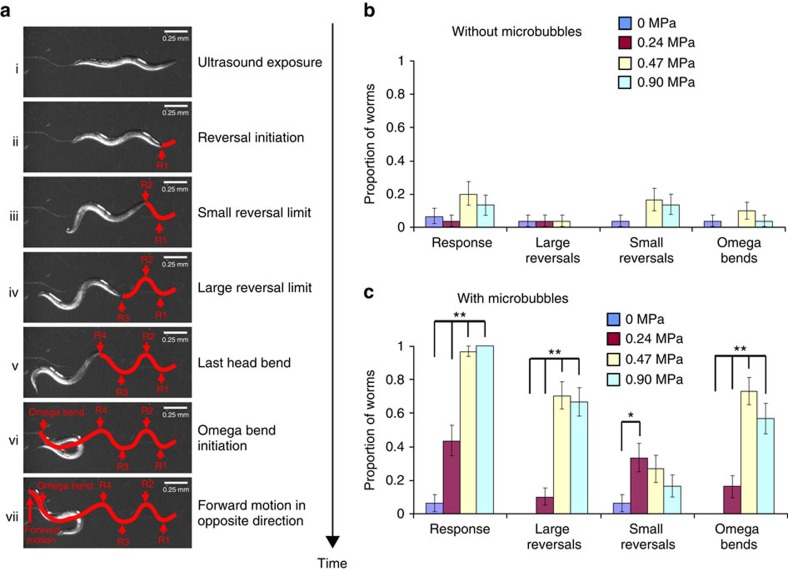
*C. elegans* behavioural responses to ultrasound in the presence of microbubbles. (**a**i-vii) Panels show an animal reversing and generating a high-angled omega bend on ultrasound stimulus. Reversals with greater than two head bends were scored as large, while those with fewer than two head bends were counted as small. Reversals and omega bends are shown with a red line overlaid on the animal tracks on the agar surface. Animal responses to ultrasound stimuli (single 10-ms pulse, 2.25 MHz) with varying peak negative pressures (**b**) without and (**c**) with microbubbles were quantified. *n*=30 for each of the conditions. Proportion of animals responding and standard error of the proportion are shown. **P<0.01 and *P<0.05 by Fisher’s exact test (two-sided).

To test whether the tiny mechanical deformations created by a single low-pressure ultrasound pulse could be amplified we included gas-filled microbubbles in our assay (Fig. 1d,e). We found that animals showed a dramatic response to ultrasound when surrounded by microbubbles (Fig. 1e). When the ultrasound wave was focused on the head of a worm surrounded by microbubbles, the animal immediately initiated a backward movement (termed ‘reversal’^28^) followed by a high-angled turn (identified as an ‘omega bend’^28^) (Figs 1e and 2a). These behaviours were scored as previously described (Methods, Fig. 2a)^28^ and are quantified as shown in Fig. 2b,c. Reversals were separated into two categories: ‘large’ reversals involving three or more head bends and ‘small’ reversals with two or less head bends (Fig. 2a)^28^. All behavioural data were analysed using a non-parametric Fisher’s exact test (two-sided), with error bars showing the standard error of the proportion. The animal’s behavioural responses were positively correlated with the pressure of the ultrasound wave in the presence of microbubbles (Fig. 2c). Importantly, using a thermocouple, we found that the presence of microbubbles did not cause any measurable increase in the ultrasound-induced temperature change at the agar surface (see Methods). Collectively, these results showed that *C. elegans* responds behaviourally to a single pulse of low-pressure ultrasound in the presence of microbubbles.

**Figure 3:**
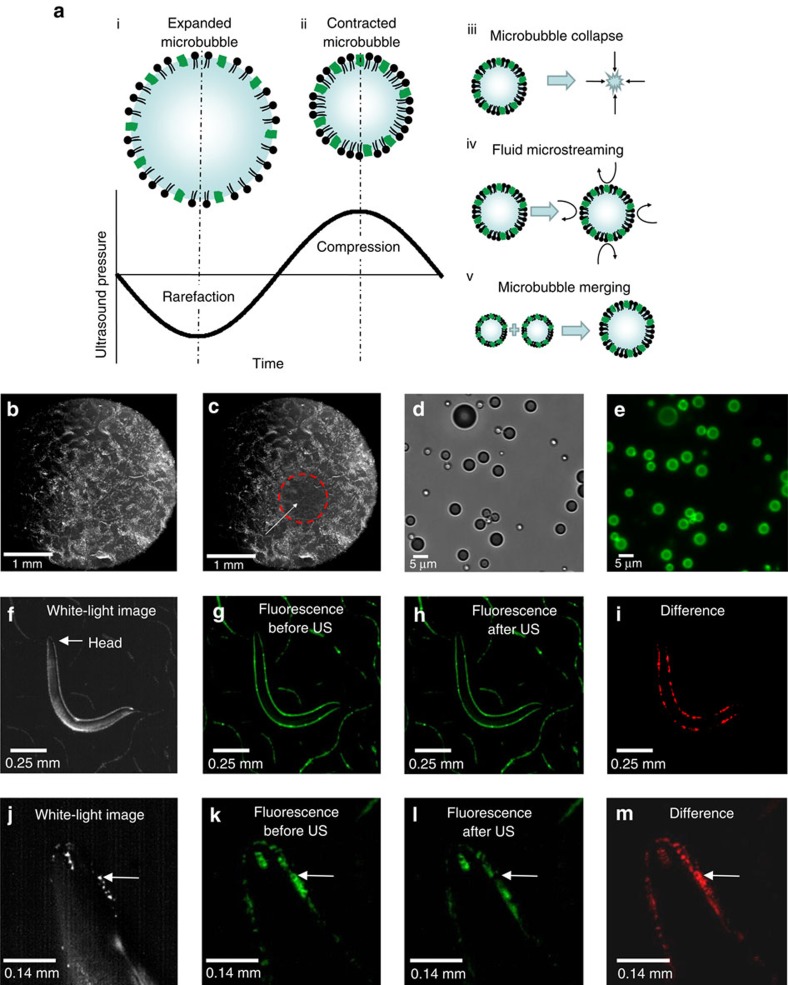
Ultrasound−microbubble interaction with *C. elegans*. (**a**) The microbubble (i) expands and (ii) contracts in size with the rarefaction (low-pressure) and compression (high-pressure) phases of the ultrasound pressure wave. This oscillation behaviour occurs at the frequency of the driving ultrasound, resulting in a variety of behaviours including (iii) microbubble collapse, (iv) fluid microstreaming and (v) merging of microbubbles. These microbubble behaviours create mechanical distortions that can propagate through the agar and the body of the animal. (**b**) Microbubbles are uniformly distributed on an agar surface and appear white. (**c**) Ultrasound stimulus (10 pulses lasting 10 ms each with a duty cycle of 1 Hz, 2.25 MHz with a peak negative pressure of 0.9 MPa) activates and destroys the microbubbles in the ultrasound focal zone of 1 mm diameter (white arrow). Microbubbles outside this focal zone (denoted by the red circle) appear undisturbed. Microbubbles were labelled with DiO, and (**d**) brightfield and (**e**) fluorescent images are shown. A whole-animal view showing (**f**) brightfield, (**g**) fluorescence before and (**h**) after ultrasound stimulus and finally, (**i**) the difference in red showing the locations of microbubble-induced mechanical deformations. A magnified view of the animal’s head in (**j**) brightfield, (**k**) before and (**l**) after ultrasound stimuli (US) and (**m**) the difference in red showing the locations of microbubble-induced mechanical deformations. The white arrow points to a large microbubble that is destroyed upon ultrasound stimulation.

To probe how microbubbles transduce the ultrasound wave and modify animal behaviour, we analysed their behaviour upon ultrasound stimulation. Previous studies have shown that the majority of the ultrasound energy propagates through water and soft tissue as a longitudinal wave with alternating compression and rarefaction phases. These two phases create pressures that are alternately higher and lower than the ambient pressure level, respectively^17^. We designed our microbubbles to respond to the mechanical deformations induced by an ultrasound pulse. We filled our microbubbles with a stabilizing mixture of perfluorohexane and air that allows the compression and rarefaction phases of the ultrasound wave to shrink and expand the microbubbles from one half to four times their original diameters^29^ in a process known as stable cavitation. This occurs at the driving frequency of the underlying ultrasound pulse^30^,^31^ (Fig. 3a). These size oscillations create mechanical deformations of the surrounding fluid^31^ as well as fluid microstreaming^32^ that can push against surfaces^32^,^33^. The microbubbles can also undergo lateral translations^34^ and complete collapse, creating large mechanical deformations that have been shown to propagate through water and tissue^31^. We found that ultrasound exposure affected the distribution of microbubbles in the focal zone on agar plates confirming these mechanical deformations (Fig. 3b,c). Next, we hypothesized that these large mechanical deformation events created by the ultrasound–microbubble interaction likely propagate from the location of the microbubbles into the body of the worm, thereby affecting behaviour. To test our hypothesis, we analysed the behaviour of microbubbles labelled with a fluorescent dye, DiO (Fig. 3d,e). We found that these microbubbles naturally distribute themselves around the animal, but are not attached to the animal’s body (Fig. 3f,g,j,k). On ultrasound stimulation, some microbubbles collapse, some merge together, while others move (Fig. 3h,l). These mechanical deformations occur all around the worm, including the worm’s head, and are specifically captured in the difference image (shown in red, Fig. 3i,m). Taken together, these results suggest that the ultrasound–microbubble interaction creates mechanical disturbances around the worm that result in increased reversal behaviour.

**Figure 4:**
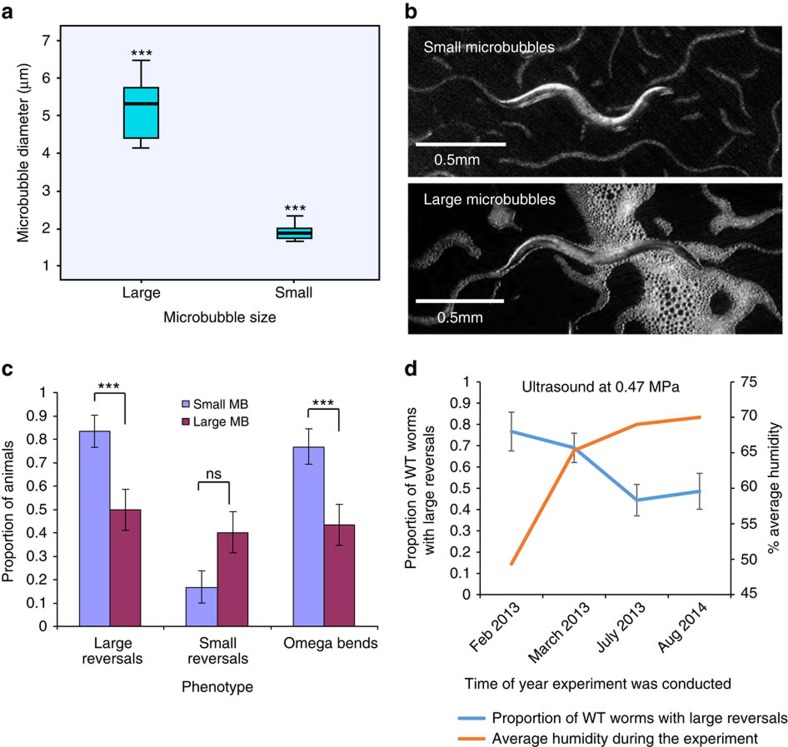
Microbubbles transduce ultrasound stimuli. (**a**) A bar and whisker plot showing the distribution of microbubbles fractionated based on their size. A one-way ANOVA test shows significance in the distribution (***P<0.001 using the LSD *post hoc* test). A mixed-size population was used for all experiments to maintain consistency. (**b**) Images showing animals incubated with small (1.5–2.5 μm; top) and large (4–6.5 μm; bottom) populations of microbubbles. (**c**) Behavioural responses of wild-type animals incubated with small and large microbubbles upon ultrasound stimulation with a single 10-ms pulse at 2.25 MHz with peak negative pressure of 0.9 MPa. Averages and standard error of the proportion are shown. ***P<0.001 using Fisher’s exact test. (**d**) A graph showing the effect of external humidity levels on animal reversal behaviour. We observed that at different times of the year the animals had different reversal behaviour in response to the same 0.47 MPa ultrasound exposure. Under low-humidity levels the animals would undergo more large reversals than under high-humidity conditions. We accounted for this variable behaviour by running a wild-type control for each of the genetically modified strains that was tested. These controls were run on the same day and under the same conditions as the tested strain. Error bars show standard error of the proportion.

The microbubble synthesis process creates a size distribution of microbubbles from 1.5 to 6.5 μm in diameter (see Methods). To better understand the effect of microbubbles from different size ranges we separated the mixed microbubbles into distinct large and small size categories based on settling time (see Methods, Fig. 4a). Both small and large microbubbles surround the animals completely (Fig. 4b). Interestingly, we found that the smaller microbubbles stimulated a significantly larger proportion of worms to generate large reversals when exposed to ultrasound, compared with larger microbubbles (Fig. 4c). To be consistent in the size distribution between different experiments we used well-mixed samples. This enabled us to ensure that worms were reproducibly exposed to the same microbubble size distribution for all experiments. We found that the proportion of wild-type animals that responded to the same ultrasound peak negative pressure (0.47 MPa) in the presence of microbubbles changed over several months. Recent work has shown that *C. elegans* detects humidity changes and modifies its locomotory behaviours^35^. We also tracked environmental conditions during the days that experiments were conducted and found that the wild-type animals’ reversal responses were related to the relative humidity level. At low humidities more animals responded, while at higher humidities fewer animals executed reversal responses (Fig. 4d). We suggest that the observed variability in wild-type behaviour is independent of the ultrasound stimulus and depends on environmental humidity. We controlled for this by testing wild-type animals along with the mutants or transgenics on the same day. We also repeated our analysis over multiple days for each genotype to assure reproducibility.

### TRP-4 ion channels sensitize neurons to ultrasound

**Figure 5:**
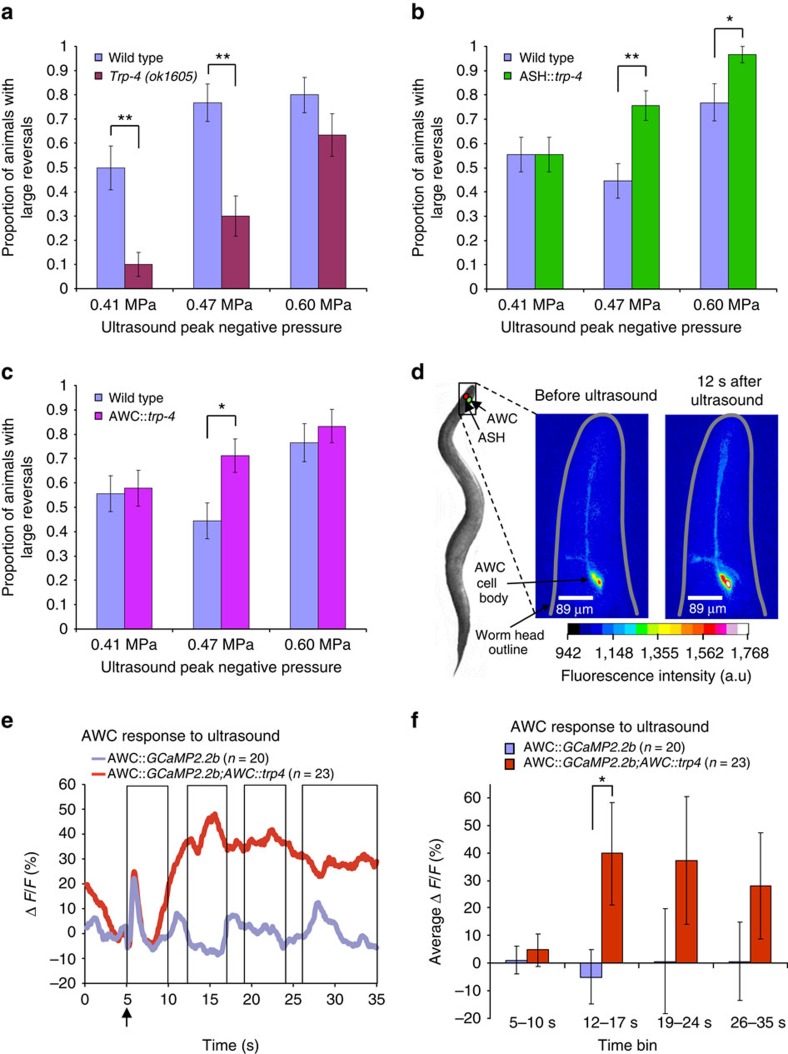
TRP-4 expression activates ASH and AWC neurons in the presence of microbubbles. (**a**) Large reversal responses to low-pressure ultrasound require the pore-forming TRP-4 channel. *n*=30 for each condition. Transgenic animals expressing TRP-4 in (**b**) ASH neurons and (**c**) AWC neurons execute more large reversals on low-pressure ultrasound stimulation (single pulse, 2.25 MHz, 10 ms). *n*>30 for each genotype and condition. The proportion and standard error of the proportion are shown in all data panels. **P<0.01, while *P<0.05 by Fisher’s exact test (two-sided). (**d**) Schematic identifying chemosensory neurons ASH and AWC in *C. elegans*. False-coloured images showing changes in GCaMP fluorescence in AWC neurons on ultrasound stimulation. Warmer colours indicate increased calcium and neural activity. (**e**) Average AWC calcium responses upon ultrasound stimulation at *t*=5 s (*AWC::GCaMP2.2b n*=20, *AWC::GCaMP2.2b;AWC::trp4 n*=23). (**f**) Average responses binned by distinct times for *AWC::GCaMP2.2b* and *AWC::GCaMP2.2b;AWC::trp4* animals. Averages and s.e.m. are shown. *P<0.05 by *t*-test (two-sided, equal variances not assumed, *AWC::GCaMP2.2b n*=20, *AWC::GCaMP2.2b;AWC::trp4 n*=23).

We hypothesized that mechanotransduction channels transduce the mechanical deformations of the ultrasound–microbubble interactions to stimulate individual neurons. Previous studies have identified TRP-4 as a stretch-sensitive, pore-forming cation mechanotransduction channel in *C. elegans*^36^,^37^. This channel is specifically expressed in a few *C. elegans* neurons, the four CEPs (CEPDL, CEPDR, CEPVL and CEPVR) and the two ADE (ADEL and ADER) dopaminergic neurons and the DVA and DVC interneurons^36^,^37^. Moreover, TRP-4 is both necessary and sufficient to generate mechanoreceptor currents in CEP neurons^36^. We tested whether TRP-4 was required to transduce the ultrasound–microbubble mechanical stimulus and modify animal behaviour. To probe ultrasound-induced behaviours we examined the effects of three different ultrasound pressure levels and found the responses to be dose dependent. We consistently found larger wild-type responses to ultrasound (see wild-type data in Figs 4d and 5a). In contrast, we found that animals lacking functional TRP-4 have reduced large reversal responses to specific peak negative pressures (0.41 and 0.47 MPa) of ultrasound stimulation, suggesting that this channel increases the probability of large reversals (Fig. 5a). However, with an ultrasound pulse at 0.6 MPa peak negative pressure, *trp-4* mutants have similar behavioural responses compared with wild type (Fig. 5a), suggesting that there is an alternate pathway that detects ultrasound at these higher pressures. Moreover, these *trp-4* mutants also show a significant increase in small reversal behaviours on ultrasound stimulation at 0.41 and 0.46 MPa (Supplementary Fig. 3a), but no change in their omega bend behaviours (Supplementary Fig. 3b). Collectively, these results suggest that TRP-4 might be activated in response to ultrasound with peak negative pressure levels less than 0.5 MPa and modifies neurons involved in generating both small and large reversals.

To test whether ultrasound sensitivity could be conferred to other neurons, we analysed the behaviour of transgenic animals misexpressing TRP-4 in specific chemosensory neurons. We tested for dose-dependent responses using three different ultrasound pressure levels. We initially misexpressed the TRP-4 channel in ASH, a well-studied polymodal nociceptive neuron^38^, whose activation leads to large reversals^39^. Consistently, we found that ASH expression of TRP-4 generated a significant increase in large reversals at ultrasound peak negative pressures of 0.47 and 0.6 MPa (Fig. 5b). Moreover, we found that these *ASH::trp-4* transgenics show a decrease in small reversals (Supplementary Fig. 3c), but not omega bends (Supplementary Fig. 3d), indicating that activating ASH via ultrasound and TRP-4 specifically modifies the neural circuits driving small- and large-reversal behaviours. Next, we tested the effects of TRP-4 misexpression on the function of the AWC sensory neurons and AWC-driven behaviour. Previous results have implied that AWC activation is correlated with an increase in the animal’s ability to generate large reversals^21^. We found that animals misexpressing TRP-4 in AWC neurons also initiated significantly more large reversals at the ultrasound peak negative pressure of 0.47 MPa (Fig. 5c), but we did not observe a change in small reversals or omega bends (Supplementary Fig. 3e,f). These results indicate that misexpressing the mechanotransduction channel, TRP-4, in ASH or AWC renders the neurons sensitive to low-pressure ultrasound, increasing the frequency of large reversals.

To test whether ultrasound could directly stimulate AWC neurons, we expressed the calcium indicator GCaMP3 (ref. 40) in these neurons and recorded their activity levels upon ultrasound exposure (Fig. 5d). Consistent with our behavioural data, we found that ultrasound stimulation in the presence of microbubbles activated AWC neurons, as indicated by an increase in the fluorescence signal from the calcium sensor. The AWC calcium responses were greatly increased in transgenic animals expressing TRP-4 specifically in AWC neurons (Fig. 5d–f, Supplementary Fig. 4a–c). We also observed that AWC calcium responses were variable, with many transients lasting more than 30 s (the duration of our recording) and a few returning back to baseline (Supplementary Fig. 4c). Variability in AWC calcium responses has also been previously observed using odour stimuli^21^. To test whether AWC responses were related to artefacts in our imaging set-up, we recorded AWC calcium in the absence of ultrasound stimulus and found no consistent responses, suggesting that AWC responded to ultrasound stimulus (Supplementary Fig. 4d,e–g). Both wild-type AWC neurons and those misexpressing TRP-4 showed a response lasting about 2–3 s immediately on exposure to a single ultrasound pulse in the presence of microbubbles. However, we also observed that AWC neurons misexpressing TRP-4 show a significant increase in their activity starting at 7 s after ultrasound exposure (*t*=12 s in Fig. 5f) and lasting for at least 5 s, which is not observed in wild-type neurons. This sustained increase in AWC calcium levels likely represents the activity of TRP-4, which could potentiate calcium entry into the neuron via other calcium channels. Interestingly, large reversals take approximately 10–20 s to complete, a time window where we also observe sustained AWC calcium activity in the *AWC::trp-4* transgenics. We suggest that the sustained AWC calcium activity observed in these *AWC::trp-4* transgenics is likely correlated with the increased frequency of large reversals generated by these animals after ultrasound stimulation. Taken together, these results show that TRP-4 channels are sensitive to low-pressure ultrasound, and ectopic expression of these channels in sensory neurons causes correlated changes in neuronal activity and behaviour.

### New roles for PVD sensory and AIY interneurons

**Figure 6:**
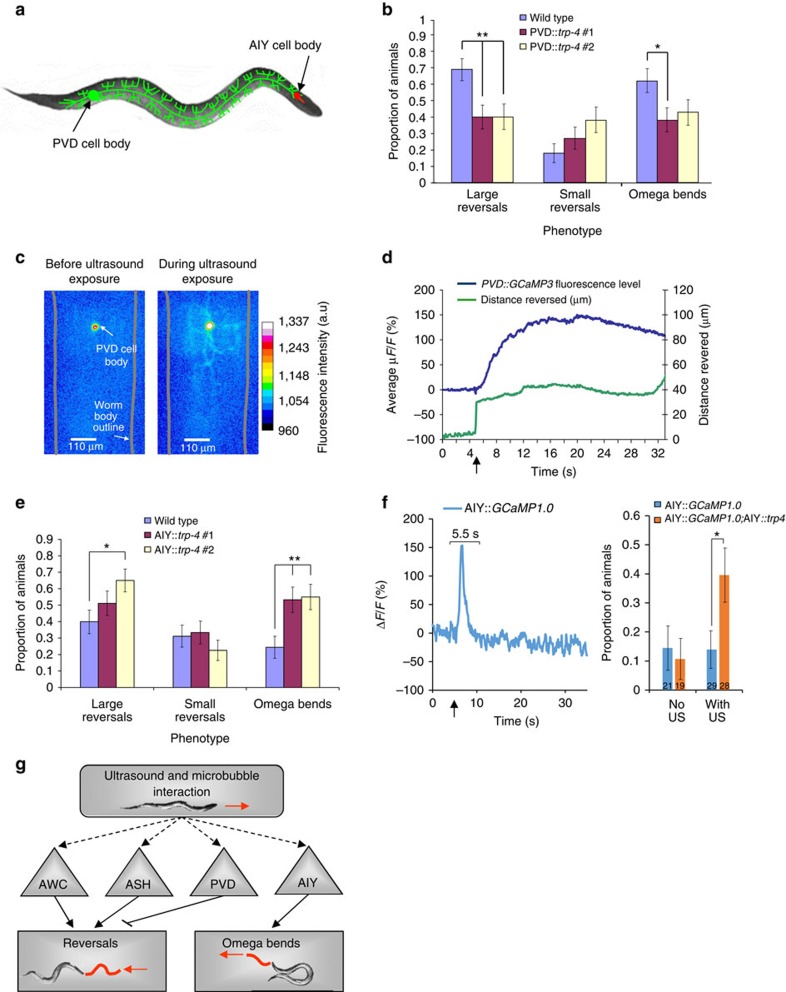
PVD neurons inhibit reversals and AIY neurons stimulate omega bends in the presence of microbubbles. (**a**) Schematic showing the PVD and AIY neurons in *C. elegans*. (**b**) Two independent transgenic strains expressing TRP-4 in PVD neurons show reduced reversals when stimulated with a single 10-ms 2.25 MHz 0.47 MPa peak negative pressure ultrasound pulse. *n*>46 for each genotype. The proportion and standard error of the proportion are shown. **P<0.01, while *P<0.05 by Fisher’s exact test (two-sided). (**c**) False-coloured images showing changes in GCaMP3 fluorescence in PVD neurons upon ultrasound stimulation. Warmer colours indicate increased calcium and neural activity. (**d**) Average PVD calcium responses (*n*=16) along with average distance moved by the animal shown as a function of time. Peak PVD response occurs when the animal has stopped moving. (**e**) Two independent transgenics expressing TRP-4 in AIY neurons show increased incidences of omega bends when stimulated with a single 10 ms 2.25 MHz 0.41 MPa peak negative pressure ultrasound pulse. *n*>40 for each genotype. (**f**) AIY calcium responses to ultrasound stimuli. A representative trace showing the ratio of change in fluorescence to the baseline is shown. Ultrasound stimulus was given at *t*=5 s and neurons that responded within a 5.5-s window after the stimulus were counted as responders. Bar graphs show % responders with and without ultrasound stimuli for *AIY::GCaMP* and *AIY::GCaMP;AIY::trp-4*. Numbers on the bars indicate the number of animals analysed in each condition. *P<0.05 by Fisher's exact test. (**g**) Schematic showing the neural circuit that responds to ultrasound stimuli amplified by microbubbles. ASH and AWC neurons promote reversals, while PVD neurons inhibit reversals. AIY neurons promote omega bends.

To test whether our method of ultrasound activation of neurons can reveal unidentified behavioural roles, we misexpressed TRP-4 in the poorly understood PVD neurons (Fig. 6a). PVD neurons have extensive, regularly spaced and non-overlapping dendritic arbours that extend throughout most of the animal, excluding the head and the neck^41^. PVD shares promoters with the FLP, IL2 and PVC neurons^42^ and so the TRP-4 proteins were expressed in multiple neurons in the *PVD::trp-4* transgenic animals. We find that expressing TRP-4 in PVD, FLP, IL2 and PVC neurons leads to a significant decrease in the animals’ reversal responses on ultrasound stimulation (two independent transgenics, Fig. 6b). We did not observe changes in FLP or other neurons in response to the ultrasound stimulus (Supplementary Fig. 5a,b). We hypothesize that misexpressing TRP-4 channels activates PVD neurons upon ultrasound stimulation, which in turn suppresses reversals. To test our hypothesis we monitored PVD neuron activity in response to ultrasound stimulation. We observed a sharp increase in PVD calcium over many seconds (Fig. 6c,d and Supplementary Fig. 6a). The initial decline is likely an artefact of the ultrasound stimulus as it was observed in both AWC and PVD recordings (Figs 5e and 6d). Interestingly, we find a strong correlation between PVD activity and animal movement. In particular, we find that PVD neurons reach their maximum response when the animal has finished reversing (Fig. 6d). Moreover, we find that PVD neurons are more likely to be activated when the animal is moving backward rather than when moving forward in response to the ultrasound stimulus (Supplementary Fig. 6b–d). These results suggest that ultrasound stimulation activates TRP-4-expressing PVD neurons and causes premature suppression of backward movement, leading to fewer reversals.

We then tested whether our approach can manipulate the function of an interneuron, whose processes do not contact the external cuticle of the animal. We misexpressed TRP-4 in AIY interneurons, which are at least 25 μm from the cuticle^19^, and analysed the behaviour of these animals upon ultrasound stimulation. Optogenetic studies have previously shown that activating AIY interneurons reduces reversals and omega bends^43^. In contrast, we find that *AIY::trp-4* transgenics are significantly more likely to initiate high-angled omega bends on ultrasound stimulation (two independent transgenics, Fig. 6e). It is possible that expressing TRP-4 in AIY neurons has altered that neuron’s function, leading to increased omega bends. Also, animals with genetically altered AIY function have been shown to have increased turns in a local search assay^21^,^28^. We found that these *AIY::trp-4* transgenics did not show any defects in local search (Supplementary Fig. S7a–c), confirming that the AIY neurons were not altered in these animals. These data suggest that AIY can initiate different behaviours based on type of stimulation, ultrasound or light.

To confirm whether ultrasound stimulus is activating AIY interneurons, we used calcium imaging. AIY neural activity is typically measured from a bulb in the AIY neurite^21^. Consistent with previous observation^21^, we found that AIY is a noisy neuron that shows a number of transients during our recordings (Supplementary Fig. 8). We collected a number of AIY recordings from wild-type animals and defined the relevant transient. We counted all animals whose neurons responded within a 5.5s time window after the ultrasound pulse as responders. Using these criteria, AIY neurons in wild-type animals did not show a significant response to ultrasound stimulus (4/29) (Fig. 6f and Supplementary Fig. 8a,b). In contrast, we observed that a significant number of AIY neurons in *AIY::trp-4* transgenics (11/28 animals) had a positive response (Fig. 6f and Supplementary Fig. 8e,f). The increased proportion of AIY responders in the *AIY::trp-4* transgenics suggests that ultrasound stimulus activates AIY interneurons. These results show that mechanical deformations from the ultrasound–microbubble interaction can penetrate at least 25 μm into the worm and influence the function of AIY interneurons. Moreover, we find that misexpressing TRP-4 can influence both reversal and omega bend neural circuitry, suggesting that our sonogenetic approach is broadly applicable for manipulating circuit activity. Further, our results show that AIY interneurons are likely to have at least three activity states with one suppressing turns^21^, one promoting forward turns (as revealed by optogenetic stimulation)^43^ and one increasing omega bends (as revealed by ultrasound stimulation). These studies validate our approach of using sonogenetics to reveal novel roles for both PVD and AIY neurons in modifying turn behaviour.

## Discussion

Our studies show that *C. elegans* neural circuits can be probed by combining low-pressure ultrasound stimulation with microbubbles that amplify the mechanical deformations. Specifically, we find that *C. elegans* is insensitive to low-pressure ultrasound but responds when surrounded by microbubbles. We find that animals missing the TRP-4 mechanosensitive ion channel have significantly reduced sensitivity to the ultrasound–microbubble stimulation, indicating that mechanosensitive ion channels play an important role in the mechanism of ultrasound stimulation. We also find that misexpressing the TRP-4 mechanosensitive ion channel in specific neurons modifies their neural activity upon ultrasound stimulation, resulting in altered animal behaviours. Specifically, misexpressing TRP-4 in ASH and AWC sensory neurons results in an increase in large reversals, while misexpressing it in PVD neurons suppresses this behaviour, a novel role for this neuron (Fig. 6g). We also define a novel role for AIY neurons in stimulating omega bend behaviour (Fig. 6g).

Our novel method provides new insights into the neural activity patterns that drive whole-animal behaviour. We note that persistent AWC neural activity might drive reversal behaviour, providing a correlation between a distinct AWC neuronal activity pattern and whole-animal behaviour. We suggest that ultrasound stimulation might activate neurons with different kinetics than what has been seen using optogenetics. For example, activating AIY interneurons using light leads to an increase in forward turns^43^, while our studies using low-pressure ultrasound indicate an alternative role for AIY interneurons in promoting and increasing frequency of omega bends. The stimulation of AIY interneurons demonstrates that this ultrasound technique can also be applied to deep internal neurons that do not contact the skin of the worm. Taken together, these results and other studies^44^ show that TRP channels can be used to manipulate neuronal functions and thus provide insight into how neural circuits transform environmental changes into precise behaviours.

To target smaller groups of neurons, the resolution of the ultrasound focal zone can be made smaller than the 1 mm diameter used in these studies. Frequencies above 2.25 MHz can produce sub-millimetre focal zone spot sizes^45^. Higher-frequency ultrasound waves with their smaller focal zones are better suited to targets that are closer to the body surface as these waves do not penetrate tissues as well^46^. One of the advantages of ultrasound is that small focal zones of just a few millimeters can be maintained noninvasively even in deep brain tissue^8^. Outside the focal zone the peak negative pressures are significantly lower and are unlikely to result in neuron activation^11^,^47^. This was seen on the agar plates where only worms that were in the focal zone responded to the ultrasound and nearby worms that were outside the focal zone did not. Another advantage of ultrasound is that this focal zone can be moved arbitrarily within the tissue to simulate different regions without any invasive procedures^8^,^47^,^48^. With an electronically steerable ultrasound beam, multiple different targets can be noninvasively manipulated either simultaneously or in rapid succession^48^. Moreover, the genetic targeting of the stretch-sensitive ion channels to individual neurons allows for targeting well below the resolution of the ultrasound focal zone.

We speculate that the use of ultrasound as a non-invasive neuronal activator can be broadly applied to decode neural circuits in larger vertebrate brains with opaque skin and intact skulls. Ultrasound waves with peak negative pressures of <1 MPa have been shown to penetrate through skull and brain tissue with very little impedance or tissue damage^49^. Our results show that low-pressure ultrasound (with peak negative pressures of 0.4–0.6 MPa) specifically activates neurons expressing the TRP-4 channel. Moreover, TRP-4 channels do not have mammalian homologues^36^,^37^; therefore, it is unlikely that expressing these channels in the mammalian brain would produce deleterious effects. We suggest that neurons in diverse model organisms misexpressing this channel can be activated by ultrasound stimulation, allowing us to probe their functions in influencing animal behaviour. Additionally, other mechanosensitive channels can be explored that may be more sensitive to mechanic deformations than TRP-4. Of particular interest are the bacterial MscL and MscS channels that have different sensitivities to membrane stretch and are selective for different ions^50^. Moreover, TRP-4 and other channels may be mutated in and around the pore region to change their ion selectivity^51^ as well as their sensitivity to mechanical stretch to broaden the utility of this method.

Furthermore, if low-pressure ultrasound stimulation by itself does not activate TRP-4-expressing neurons, we demonstrate that the mechanical signals can be amplified by gas-filled microbubbles. Perfluorohexane microbubbles are well-established for use as ultrasound contrast agents *in vivo*^52^ and can be administered intravenously to circulate throughout the vertebrate body including the brain^53^,^54^,^55^,^56^. They can remain active for up to 60 min^57^, providing a time window where they could be used safely to amplify the ultrasound stimulus and manipulate neural activity. Microbubbles have been shown to undergo inertial cavitation when exposed to ultrasound with peak negative pressure of 0.58 MPa and higher^58^. Using ultrasound pressure levels lower than this will prevent damage to the brain from the microbubble–ultrasound interaction. Moreover, we used a third of the number of microbubbles that has been previously used to successfully image the mouse brain^59^, showing that the required microbubble dose would not be prohibitive for *in vivo* administration. Our experiments show that in the presence of microbubbles the low-pressure ultrasound stimulated the deep AIY interneurons expressing TRP-4. This result enables us to estimate the distances at which the mechanical deformations from the ultrasound–microbubble interaction can effectively penetrate into brain tissue from the vasculature. The *C. elegans* cuticle is 0.5 μm thick^60^ and the AIY interneurons are 25 μm from the cuticle^19^, indicating that the mechanical deformations travelled at least 25.5 μm into the worm. In contrast, the mammalian blood–brain barrier is 0.2 μm thick^61^ and the average distance of a neuron from a capillary is less than 20 μm (ref. 62). These distances are well within the range of our sonogenetic approach. With the data presented in this paper, we offer a novel, non-invasive approach to activate genetically targeted neurons using low-pressure ultrasound stimulation.

## Methods

### Ultrasound and microscopy set-up

A schematic of the ultrasound and microscopy set-up is shown in Fig. 1a and is previously described^63^. The single 10-ms, 2.25-MHz sine wave ultrasound pulse was generated with a submersible 2.25 MHz transducer (V305-Su, Panametrics, Waltham, MA) using a waterproof connector cable (BCU-58-6W, Panametrics). The resulting sound field was quantified using a needle hydrophone (HNP-0400, Onda Corporation, Sunnyvale, CA). An arbitrary waveform generator (PCI5412, National Instruments, Austin, TX) controlled by a custom-designed programme (LabVIEW 8.2, National Instruments) was used to create the desired ultrasound pulse. The peak negative pressure of the ultrasound pulse was adjusted from 0 to 0.9 MPa using a 300-W amplifier (VTC2057574, Vox Technologies, Richardson, TX). Ultrasound attenuation though the plastic and agar was found to be minimal.

White light illumination was achieved by reflecting light from an external light source up at the petri dish using a mirror mounted at 45°. Behaviour was captured using a high-speed camera (FASTCAM, Photron, San Diego, CA). Fluorescent images were collected using a Nikon 1-FL EPI-fluorescence attachment on the same set-up as described. GCaMP imaging was performed using a × 40 objective and the images were captured using a Quanti-EM 512C camera (Photometrics, Tucson, AZ).

The petri dish was held at the air–water interface with a three-prong clamp mounted to an XYZ micromanipulator stage, which allows the dish to be scanned in the XY plane while maintaining a constant Z distance between the objective and ultrasound transducer. This alignment positioned the agar surface in the focal zone of the ultrasound wave.

### Microbubble synthesis

Microbubbles were made using a probe sonication technique as described^64^. Briefly, the lipids were dissolved in chloroform and mixed together in the previously described molar ratios in a glass vial^64^. The chloroform was evaporated under an argon stream, allowing the lipids to form a layer along the sides of the glass vial. The choice of lipids is essential for tuning the behaviour of microbubbles since they can affect the stiffness of the monolayer that surrounds and protects the microbubbles. The formulation used here has a stabilizing lipid monolayer consisting of distearoyl phosphatidylcholine (DSPC, Avanti Polar Lipids Inc., Alabaster, AL), distearoyl phosphatidylethanolamine-methyl polyethylene glycol (mPEG-DSPE 5k, Layson Bio Inc., Arab, AL) and DiO (Biotium Inc., CA) in 85:13:2 molar ratio. The lipids were rehydrated in 500 μl of PBS. The headspace of the vial was filled with a perfluorohexane (Sigma-Aldrich, St. Louis, MO) and air gas mixture. A probe sonicator tip was then placed just below the surface of the PBS and run at maximum power for 3 s to cause the spontaneous formation of the lipid-coated microbubbles. The gas core of the microbubbles consisted of the perfluorohexane and air mixture which was designed to attain stability under atmospheric pressure. Microbubbles were fractionated based on size by their settling time (Fig. 4a). We chose a mixed size distribution of microbubbles from the manufacturing process to maintain uniformity across all the experiments. The microbubbles were shown to be stable on agar plates sealed with parafilm for up to 24 h.

### Behavioural assays

A 15 μl solution of microbubbles at a density of 3.8 × 10^7^/ml was added to an empty nematode growth media 2% agar plate. The microbubbles were left on the plate for 20 min to ensure absorption of the liquid leaving the microbubbles on the surface. Well-fed young adult worms were placed on the plate and were corralled into the small microbubble area using a filter paper soaked in copper sulphate solution (200 mM). The worms were allowed to crawl around for 10 min before being stimulated by ultrasound. The agar plate was moved to localize the animal in the ultrasound focal zone where it was stimulated with one pulse. Resulting reversal and omega bend behavioural responses were recorded. The ultrasound pulse regime is described above. Reversals with fewer than two head bends were identified as small (Fig. 2aiii), while those with more than two were counted as large (Fig. 2aiv). High-angled turns that lead to a significant change in the direction of an animal’s movement were identified as omega bends (Fig. 2avi)^28^. Each animal was only exposed to a single ultrasound pulse. Since previous work has shown that *C. elegans* detects changes in humidity and modifies its locomotory behaviours^35^, we controlled for humidity effects by testing wild-type animals daily to ensure stable reversal behaviour and repeating our analysis over multiple days for each genotype. Behavioural data were collected on three separate days to ensure reproducibility. The data from all three days were combined for the final statistical analysis. Significance and the relevant comparisons are shown in each figure.

Local search behaviour^28^ was used to test whether AIY functionality was affected in the *AIY::trp-4* transgenics. Briefly, day 1 adults were moved from food plates to a food-free plate and their behaviour was scored as indicated. Reversals were scored as small (fewer than two head bends) or large (more than two head bends). Four animals were scored for each genotype. Data were collected over 2 days and combined. *AIY::trp-4* transgenics executed normal local search behaviour (Supplementary Fig. 7).

### Imaging

Transgenic animals expressing GCaMP in specific neurons were corralled into a small area using a filter paper soaked in copper solution (as described above). The acetylcholine agonist and paralytic, tetramisole^65^, was used at a concentration of 1.3 mM to paralyse the animals to facilitate recording neural activity. Anaesthetized animals were surrounded by a solution of microbubbles and stimulated using ultrasound peak negative pressures as described above. Fluorescence was recorded at 10 frames s^−1^ using an EMCCD camera (Photometrics, Quant-EM) and resulting movies were analysed using Metamorph software (Molecular Devices)^21^. Briefly, a fluorescence baseline was calculated using a 2-s window from *t*=3 to *t*=5 s before ultrasound exposure. The ratio of change in fluorescence to baseline fluorescence was plotted in all graphs using custom MATLAB scripts^21^. For imaging PVD neurons, the concentration of the tetramisole was reduced to 1 mM, which allowed these animals greater movement. For imaging AIY interneurons, two doses of tetramisole at 3.9 mM were used to restrict animal movement. Here, we recorded fluorescence from the AIY process and the increased tetramisole enabled the animal to be paralysed. Their motion and corresponding fluorescent intensity changes were captured and analysed using Metamorph software. For all imaging experiments, one neuron was imaged in each animal and the animal was only used once. Imaging data were collected on three separate days to ensure reproducibility. The data from all three days were combined for the final statistical analysis.

### Molecular biology and transgenic animals

All *C. elegans* strains were grown under standard conditions as described^66^. Cell-selective expression of TRP-4 was achieved by driving the full-length cDNA under *odr-3* (AWC)^67^, *sra-6* (ASH)^68^, *des-2* (PVD and FLP)^42^ and *ttx-3* (AIY)^69^ promoters. Germline transformations were obtained using the methods previously described^70^. Complete information for all strains is listed in Supplementary Table 1.

### Temperature measurements

Temperature was measured in the ultrasound focal zone on the surface of the agar using a High Precision Digital RTD/Thermocouple Thermometer/Data Logger (model number DP9602) along with a Fine Tip TJ Probe, both from Omega (Stamford, CT). The miniature probe was 2.4 mm long by 0.5 mm in diameter (Supplementary Fig. 2). These small dimensions are within the spot size of the ultrasound focal zone and close to the dimensions of the worms themselves. The small dimensions, and correspondingly small mass of the probe, helped to reduce the effect of the probe itself removing heat from the ultrasound focal zone, making our temperature measurements as accurate as possible. The tip was placed in the focal zone of the ultrasound on the agar surface both with and without the presence of microbubbles. The agar surface was ensonified with the same ultrasound pulse used in the experiments. We observed that both with and without the presence of microbubbles an ultrasound peak negative pressure of 0.7 MPa caused a temperature increase of less than 0.1 °C.

### Statistical analysis

Data were analysed using SPSS software v22 (IBM, NY). The sample sizes used in the behavioural experiments were chosen to detect effect sizes of at least 15% with a power of 0.80. All behavioural data were analysed using a non-parametric Fisher’s exact test (two-sided). The sample sizes used in the imaging experiments were chosen to detect effect sizes of at least 20% with a power of 0.80. Animals were excluded from the study if they showed visible signs of injury or disorder, and measures used to determine an animal’s health were established prior to the study. Animals were randomly chosen from large breeding populations of the different genotypes for each of the three replicates and analysed on different days. The observer was not blind to the genotype of the group being tested. The populations used for imaging data (*t*-test) and microbubble size distribution (one-way ANOVA) had adequate normal distribution to justify the use of those tests.

## Additional information

**How to cite this article:** Ibsen, S. *et al.* Sonogenetics is a non-invasive approach to activating neurons in *Caenorhabditis elegans*. *Nat. Commun.* 6:8264 doi: 10.1038/ncomms9264 (2015).

## Supplementary Material

Supplementary Information Supplementary Figures 1-8 and Supplementary Table 1
