# Supplementary material for: Sonogenetics is a non-invasive approach to activating neurons in Caenorhabditis elegans
Source: Nat Commun. 2015 Sep 15;6:8264. doi: 10.1038/ncomms9264 (PMC4571289; doi:10.1038/ncomms9264)
Supplement: Supplementary Information — Supplementary Figures 1-8 and Supplementary Table 1 [file ncomms9264-s1.pdf]

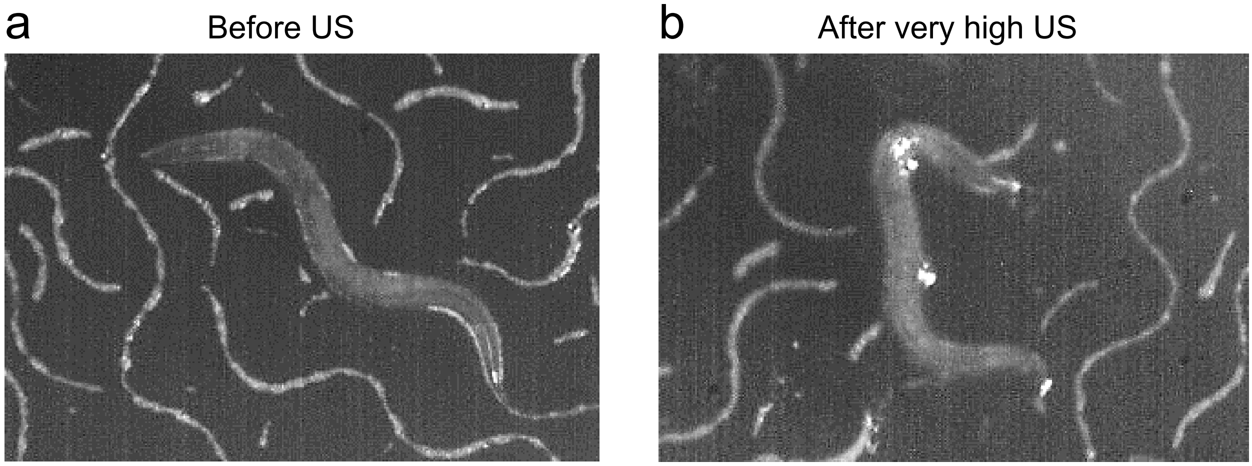

Supplemental Figure 1. Damage to worms through multiple exposures to high peak negative pressure ultrasound in the presence of microbubbles. (**a**) The worm displays a normal curved sinusoidal body position before exposure to the ultrasound. (**b**) After exposure to 10 pulses of 0.9MPa peak negative pressure ultrasound with a 1 Hz repetition rate the worm displays abnormalities in maintaining a normal body position and locomotion behavior is inhibited indicating damage has occurred.

a b

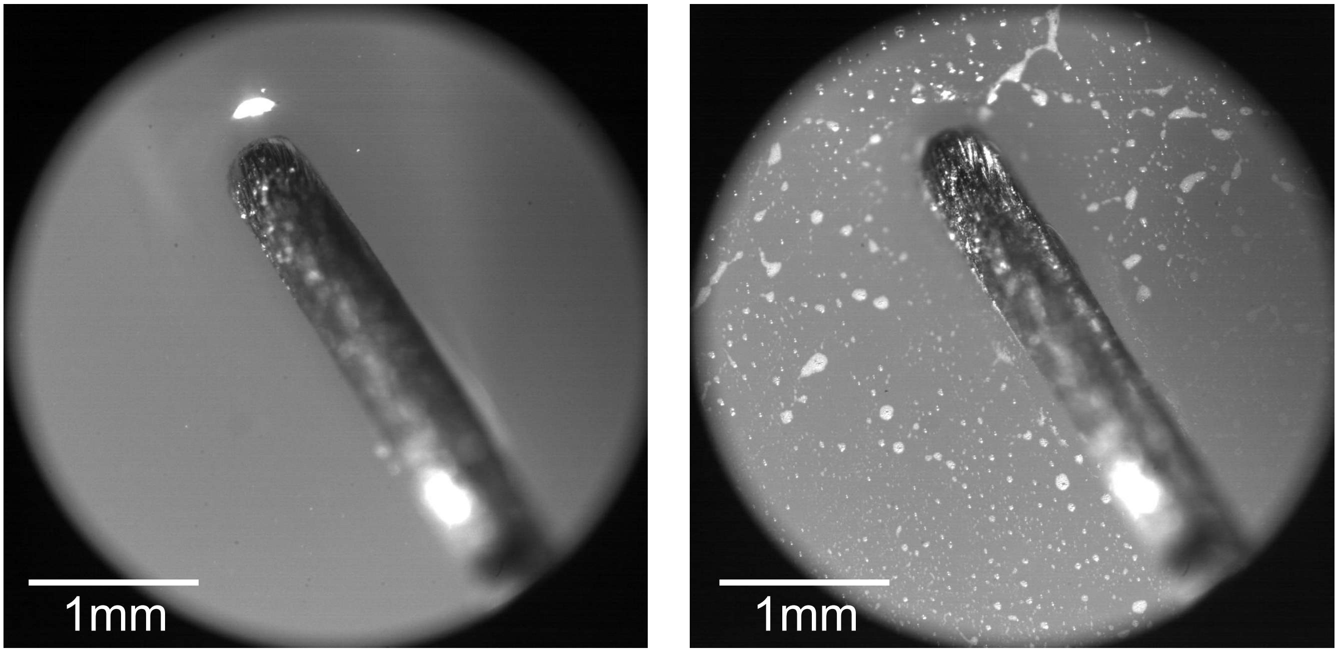

Supplementary Figure 2: Thermocouple used for measuring temperature increases on agar surfaces. Images showing the probe touching the agar surface (**a**) without and (**b**) with microbubbles.

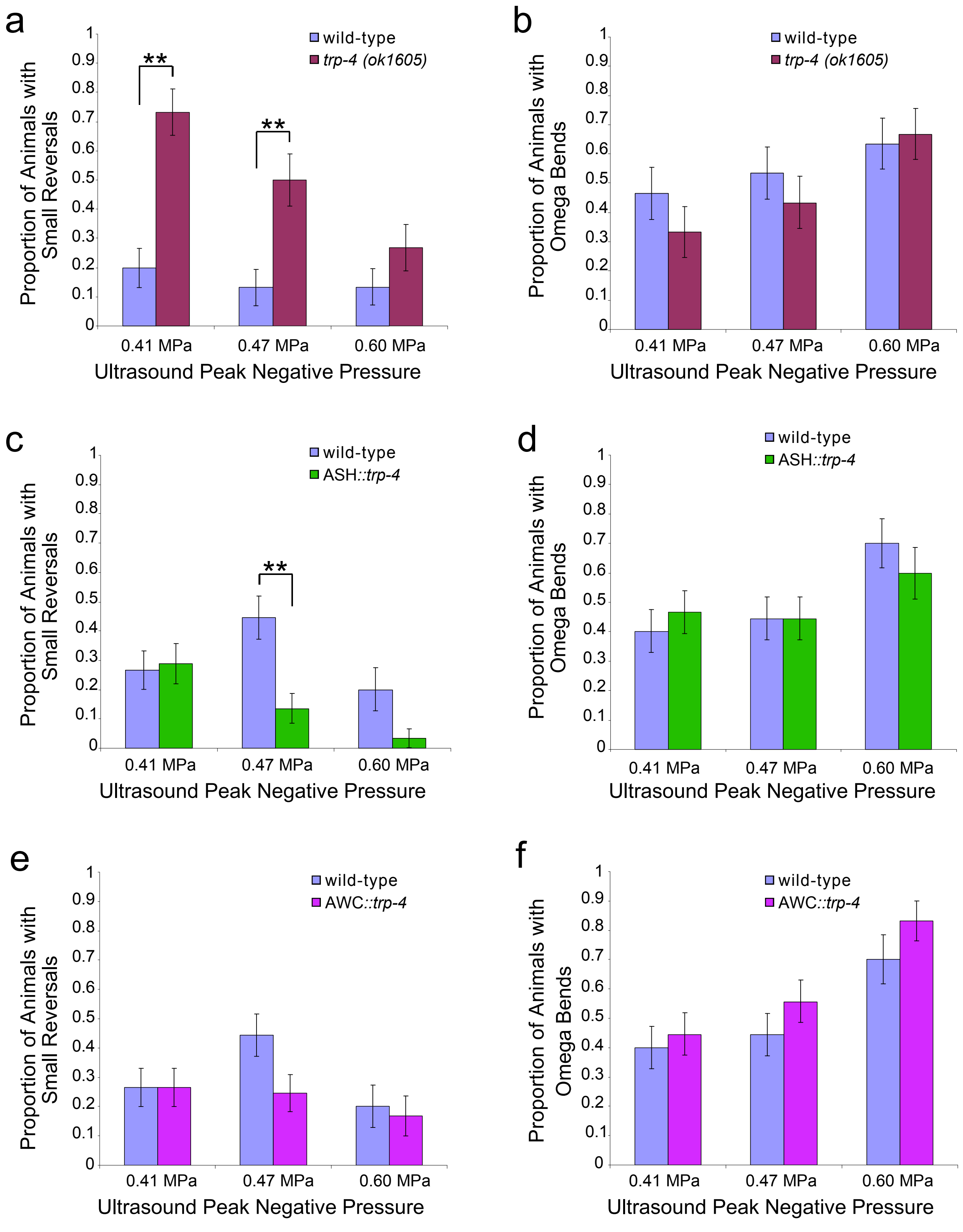

Supplementary Figure 3: Small reversal and omega bend responses to ultrasound stimuli in the presence of microbubbles. *trp-4* mutants have altered number of (**a**) small reversals, but not (**b**) omega bends when compared to wild-type animals. Transgenics expressing trp-4 in ASH neurons also exhibit fewer numbers of (**c**) small reversals, but not (**d**) omega bends. AWC::trp-4 animals do not have any significant differences in their (**e**) small reversal or (**f**) omega bend responses upon ultrasound stimulation. Proportions and standard error of the proportion are shown. ** $p < 0.01$ by Fisher's exact test.

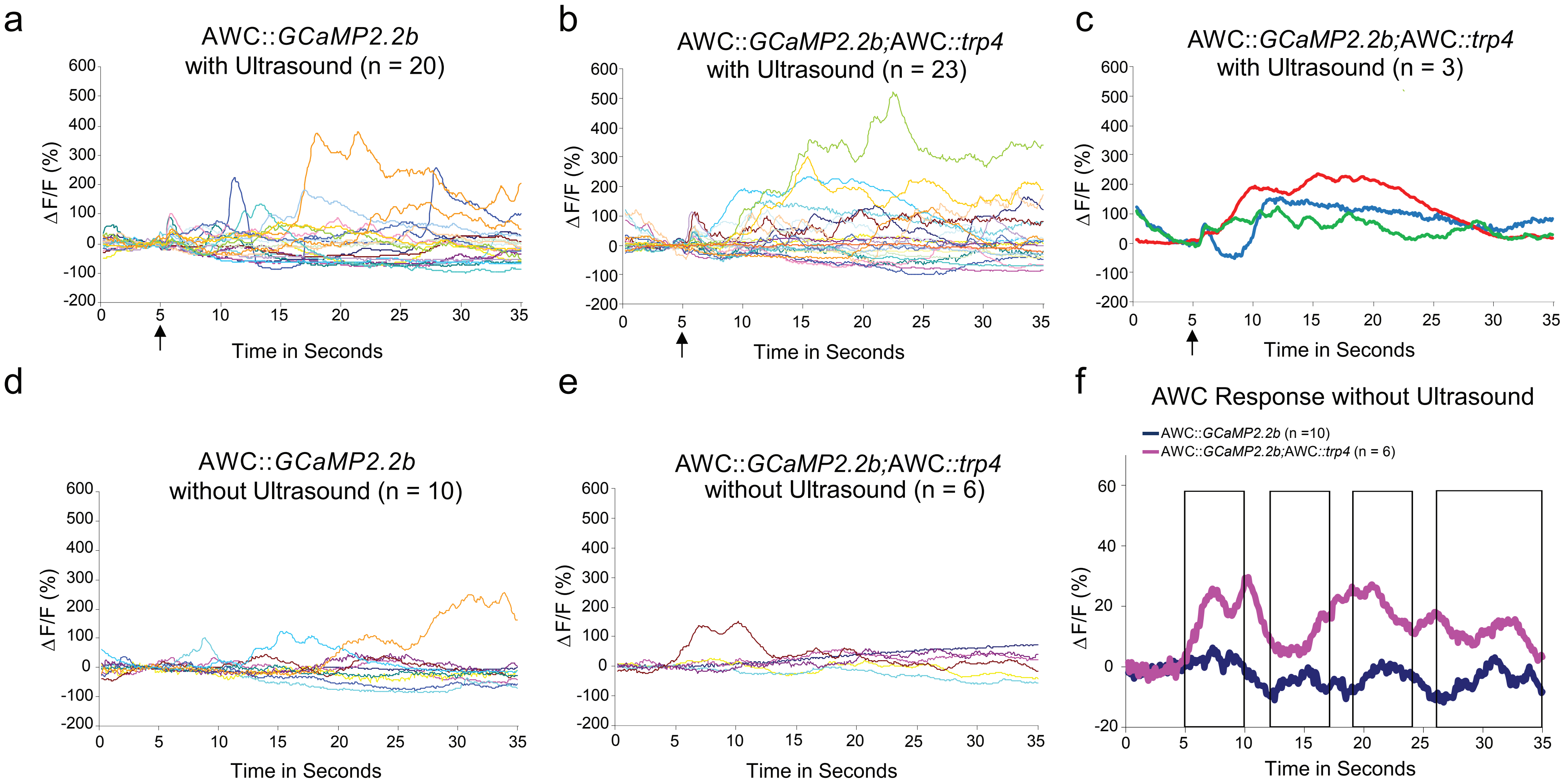

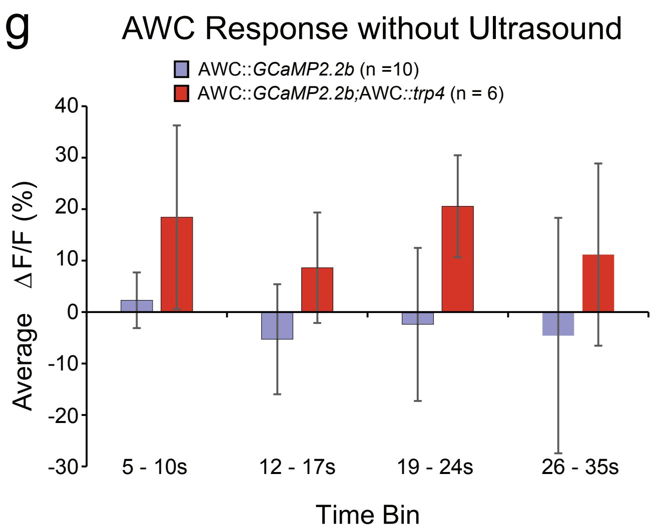

Supplementary Figure 4: AWC calcium responses to ultrasound stimulus. Ratio of change in fluoresence to baseline fluorescence in AWC neurons expressing the caclium sensor,GCaMP2.2b (n=20) (**a**) and *trp-4* specifically in AWC neurons (n=23) (**b**). Of these 23 animals tested, 3 reached the baseline (**c**). AWC calcium responses without ultrasound stimulus in wild-type (n=10) (**d**) and *AWC::trp-4* transgenics (n=6) (**e**). Each color represents the response of an individual neuron to ultrasound stimulus presented at t=5seconds. (**f**) Average AWC calcium responses without ultrasound stimulus in wild-type and *AWC:;trp-4* transgenics. (**g**) Average AWC calcium data binned similarly to the data shown in Fig. 5f. Microbubbles are included in all AWC calcium recordings.

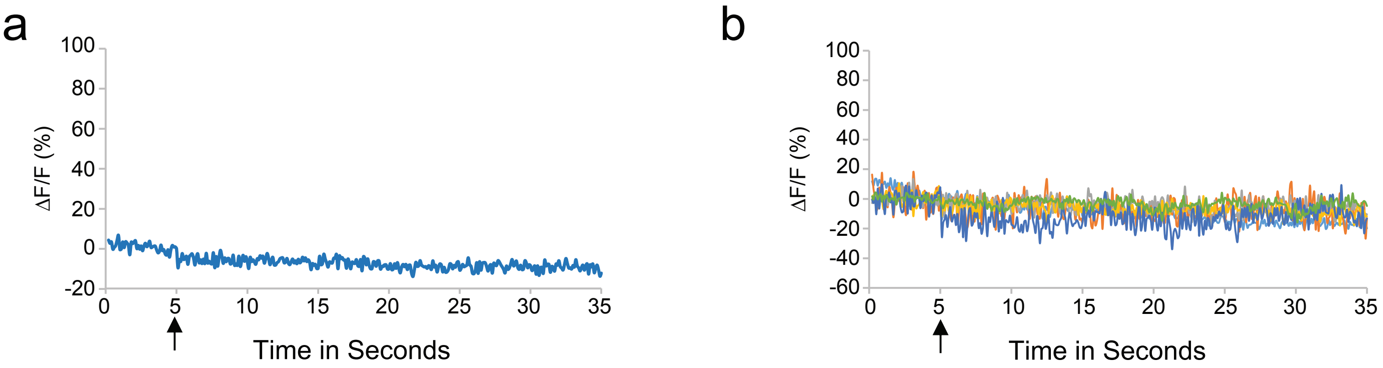

Supplementary Figure 5 FLP neurons do not respond to ultrasound **(a)** Average of 6 different FLP GCaMP responses to the ultrasound stimulus presented at t = 5s. No response was observed. **(b)** The 6 individual FLP GCaMP traces shown in panel a. Microbubbles are present in all FLP recordings.

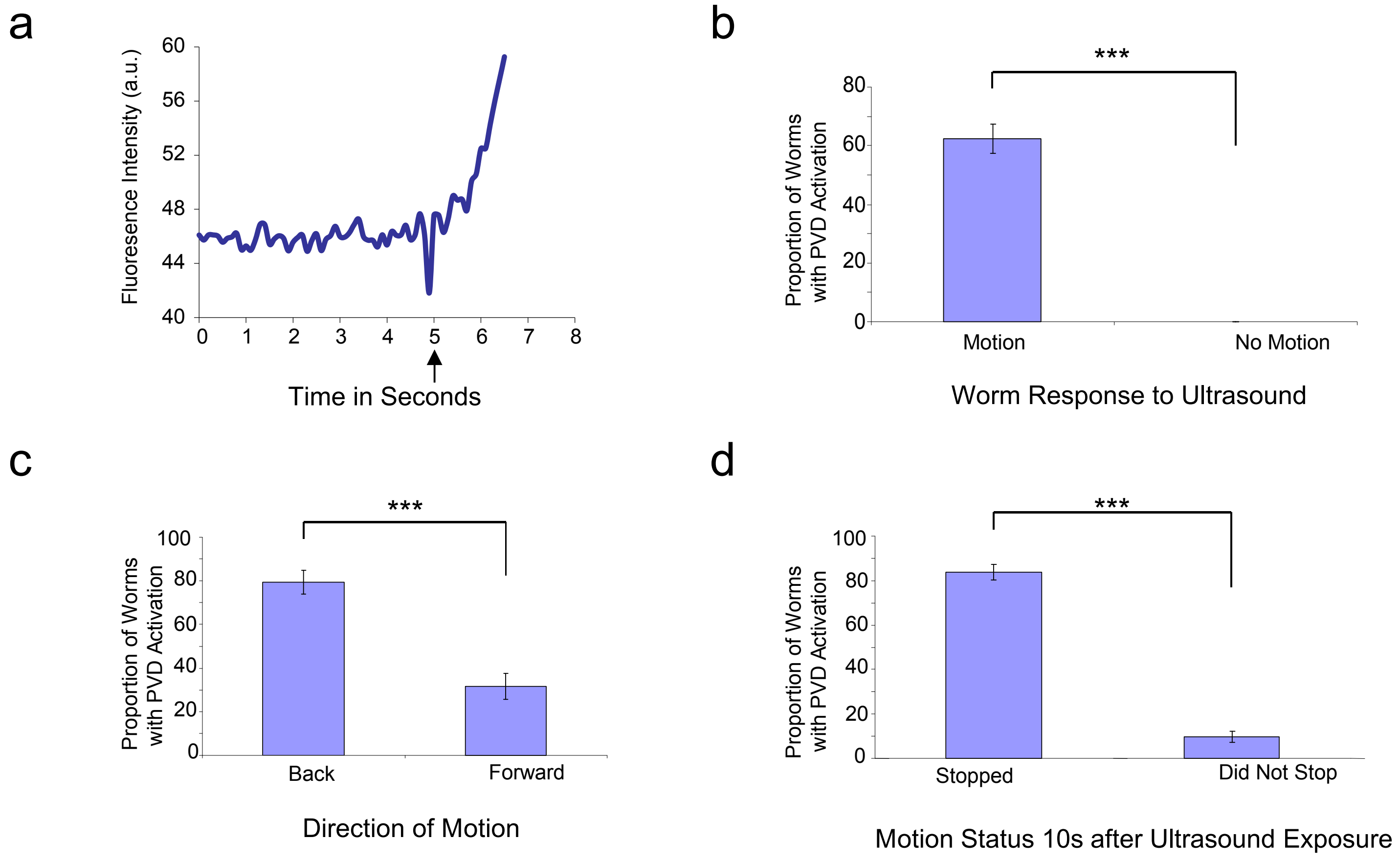

Supplementary Figure 6. PVD responses depend on worm movement. (a) Expanded view of average PVD trace in response to ultrasound and microbubbles shown in Fig 6d. The animal was stimulated with a single ultrasound pulse at t = 5s. There was an immediate decrease in fluorescence, which was then followed by a rapid increase. PVD activity is strongly correlated with **(b)** movement (n = 89), **(c)** in the backward direction (Backward n = 25, forward n = 16) and **(d)** in animals that stopped (stop or slow down n = 22, not stopping or slowing down n =19). Proportions and standard error of the proportion are shown with *** indicating p < 0.001 by Fisher' exact t-test.

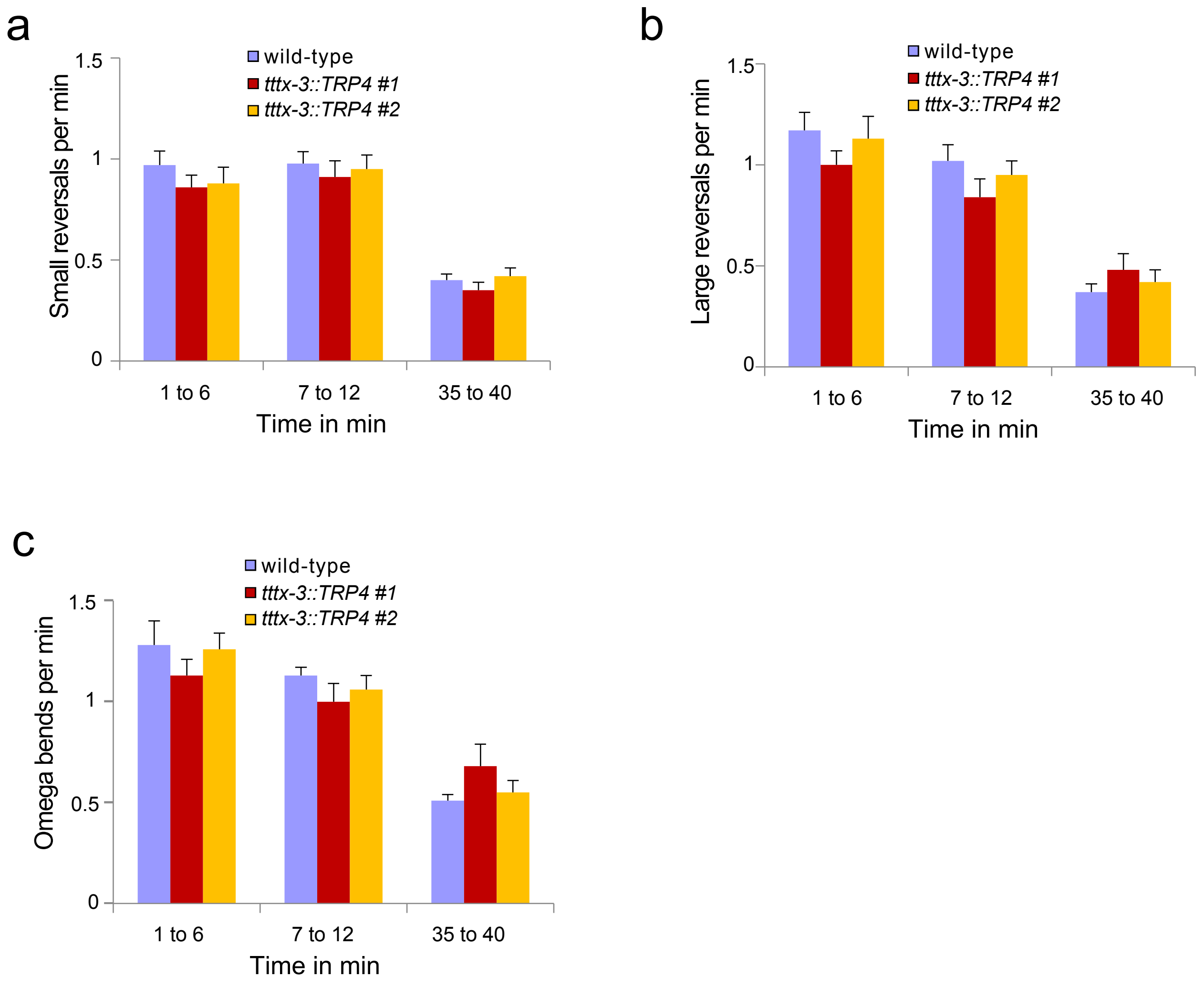

Supplementary Figure 7. AIY transgenic have normal local search. Animals were moved from food to a food-free plate and their reversals and omega bends were quantified. The two AIY::trp-4 transgenics executed normal number of small reversals (**a**), large reversals (**b**) and omega bends (**c**).

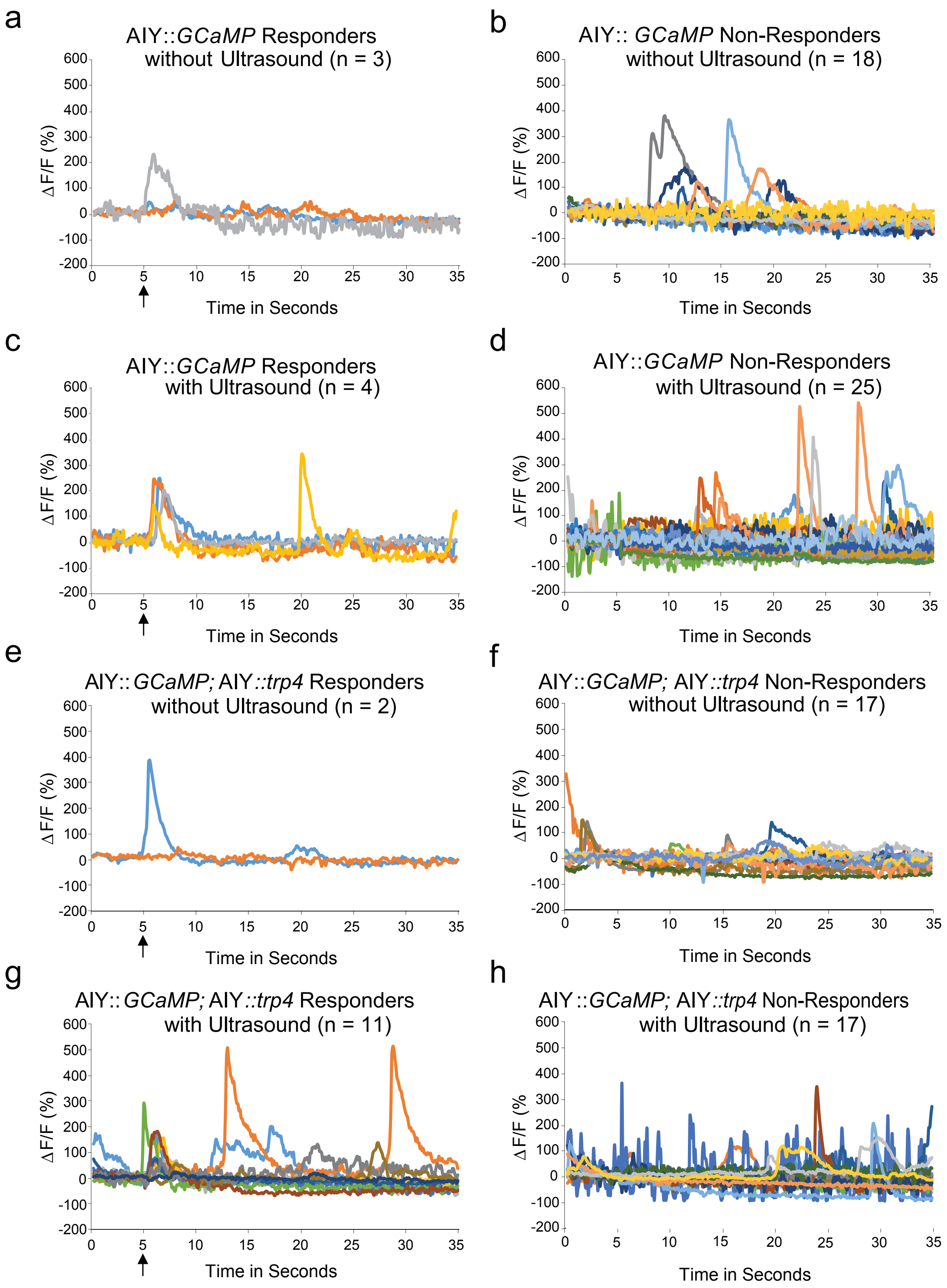

Supplementary Figure 8. AIY responses to ultrasound. (a-d) Ratio of change in fluorescence in the AIY neurite without (**a**,**b**) and with ultrasound (**c**,**d**). Neurons that responded in a 5.5 second window around t=5 seconds are shown in (a,c) and those that did not are in (**b**,**d**). Ultrasound stimulus was presented at t=5 seconds in (**c**,**d**). (**e-h**) Ratio of change in fluoresence to baseline fluoresence in the AIY neurite expressing trp-4 in AIY interneurons specifically without (**e**,**f**) and with ultrasound (**g**,**h**). Neurons that responded in the same 5.5 second window around t=5 seconds are shown in (**e**,**g**) and those that did not are shown in (**f**,**h**). Each colored trace represents data from a single neuron recorded once.

**Table S1:** Table showing list of all strains and their genotypes			
**Strain**	**Genotype**	**Description**	**Location in paper**
N2	wild-type	WT	Figure 1 C, E; Figure 2 A-C; Figure 3 F-M; Figure 4 B-D; Figure 5 A-C; Figure 6 B, E; Figure S3 A-F; Figure S6 A, B; Figure S7 A-C
VC1141	*trp-4(ok1605)*	*trp-4* mutant	Figure 5 A; Figure S3 A-B
IV133	*ueEx71 [sra-6::trp-4, elt-2::gfp]*	ASH expression of *trp-4* in wildtype background	Figure 5 B; Figure S3 C-D
IV157	*ueEx85 [odr-3::trp-4, elt-2::gfp]*	AWC expression of *trp-4* in wildtype background	Figure 5 C; Figure S3 E-F
CX10536	*kyEx2595 [str-2::GCaMP2.2b, unc-122::gfp]*	AWC imaging line in wildtype background	Figure 5 E-F; Figure S4 A, D, F, G
IV344	*ueEx219 [odr-3::trp-4, unc-122::rfp], kyEx2595 [str-2::GCaMP2.2b, unc-122::gfp]*	AWC imaging line with *trp-4* expressed in AWC	Figure 5 D-F; Figure S4 B, C, E-G
IV242	*ueEx150 [des-2::trp-4; elt-2::gfp #3]*	PVD expression of *trp-4* in wildtype background	Figure 6 B
IV243	*ueEx151 [des-2::trp-4; elt-2::gfp #4]*	PVD expression of *trp-4* in wildtype background	Figure 6 B
IV219	*ueEx134 [des-2::GCaMP3, unc-122::rfp]*	PVD and FLP imaging line in wildtype background	Figure 6 C-D; Figure S6 A-D
IV494	*ueEx307 [ttx-3::trp-4; elt-2::gfp #3]*	AIY expression of *trp-4* in wildtype background	Figure 6 E, Figure S7 A-C
IV495	*ueEx308 [ttx-3::trp-4; elt-2::gfp #4]*	AIY expression of *trp-4* in wildtype background	Figure 6 E, Figure S7 A-C
CX8554	*kyEx1489 [ttx-3::GCaMP1.0, unc-122::gfp]*	AIY imaging line in wildtype background	Figure 6F, Figure S8 A-D
IV646	*kyEx1489[ttx-3::GCaMP1.0, unc-122::gfp]; ueEx440[ttx-3::trp-4, unc-122::rfp]*	AIY imaging line with *trp-4* expressed in AIY	Figure 6F, Figure S8 E-H
